# Insights of early feeding regime supplemented with glutamine and various levels of omega-3 in broiler chickens: growth performance, muscle building, antioxidant capacity, intestinal barriers health and defense against mixed *Eimeria spp* infection

**DOI:** 10.1080/01652176.2024.2373287

**Published:** 2024-07-03

**Authors:** Asmaa T.Y Kishawy, Reham A. Abd El-Wahab, Fatma Eldemery, Mona Mohammed I. Abdel Rahman, Saleh Altuwaijri, Rasha M.M. Ezz-Eldin, Ehab M. Abd-Allah, Shimaa Zayed, Zohair S. Mulla, Rasha B. El Sharkawy, Shereen Badr, Wessam Youssef, Doaa Ibrahim

**Affiliations:** a Department of Nutrition and Clinical Nutrition, Faculty of Veterinary Medicine, Zagazig University, Zagazig, Egypt; b Biochemistry Department, Animal Health Research Institute (AHRI), Mansoura Branch, Agriculture Research Center (ARC), Giza, Egypt; c Department of Hygiene and Zoonoses, Faculty of Veterinary Medicine, Mansoura University, Mansoura, Egypt; d Department of Parasitology, Faculty of Veterinary Medicine, Zagazig University, Zagazig, Egypt; e Department of Pathology and laboratory diagnosis, College of Veterinary Medicine, Qassim University, Buraidah, Saudi Arabia; f Department of Biochemistry, Faculty of Veterinary Medicine, Zagazig University, Zagazig, Egypt; g Veterinary Educational Hospital, Faculty of Veterinary Medicine, Zagazig University, Zagazig, Egypt; h Department of Public Health, College of Veterinary Medicine, King Faisal University, Al-Ahsa, Saudia Arabia; i Department of Clinical Pathology, Zagazig Branch, Animal Health Research Institute (AHRI), Agriculture Research Center, Zagazig, Egypt; j Department of Clinical Pathology, Animal Health Research Institute (AHRI), Mansoura Branch, Agricultural Research Center (ARC), Giza, Egypt; k Department of Biotechnology, Animal Health Research Institute (AHRI), Giza, Egypt

**Keywords:** Post hatch feeding, glutamine, omega-3, tight junction, coccidiosis

## Abstract

Early nutritional management approach greatly impacts broilers’ performance and resistance against coccidiosis. The current study explored the impact of post-hatch feeding with a combination of glutamine (Glut) and different levels of omega-3 on broiler chickens’ growth performance, muscle building, intestinal barrier, antioxidant ability and protection against avian coccidiosis. A total of six hundred Cobb 500 was divided into six groups: first group (fed basal diet and unchallenged (control) and challenged (negative control, NC) groups were fed a basal diet without additives, and the other groups were infected with *Eimeria spp* and supplemented with 1.5% Glut alone or with three different levels of omega-3 (0.25, 0.5 and 1%) during the starter period. Notable improvement in body weight gain was observed in the group which fed basal diet supplemented with glut and 1% omega 3 even after coccidia infection (increased by 25% compared challenged group) while feed conversion ratio was restored to control. Myogeneis was enhanced in the group supplemented with Glut and omega-3 (upregulation of myogenin, MyoD, mechanistic target of rapamycin kinase and insulin like growth factor-1 and downregulating of myostatin genes). Groups supplemented with Glut and higher levels of omega-3 highly expressed occluding, mucin-2, junctional Adhesion Molecule 2, b-defensin-1 and cathelicidins-2 genes. Group fed 1% Glut + omega-3 showed an increased total antioxidant capacity and glutathione peroxidase and super oxide dismutase enzymes activities with reduced levels of malondialdehyde, reactive oxygen species and H_2_O_2_. Post-infection, dietary Glut and 1% omega-3 increased intestinal interleukin-10 (IL) and secretory immunoglobulin-A and serum lysozyme, while decreased the elevated inflammatory mediators comprising interleukin IL-6, tumor necrosis factor-alpha, nitric oxide (NO) and inducible NO synthase. Fecal oocyst excretion and lesions score severity were lowered in the group fed 1% Glut and omega 3. Based on these findings, dietary Glut and omega-3 supplementation augmented restored overall broilers’ performance after coccidial challenge.

## 1. Introduction

The prerequisite to achieve and sustain an improvement in poultry production is the motivating force behind the novel advances in poultry nutrition and there has been concurrent modification in the feeding and nutrition of commercial poultry that starting from the day of the hatch (Ravindran and Abdollahi 2021). The first week of life is the most critical period in the broilers’ life, its digestive and immune systems are still immature, that has a great impact on its welfare, growth performance and health as it matures (Panda et al. 2015). Moreover, there is a strong correlation between body weight on day 7 and body weight at slaughter especially the first week accounts for 20–25% of the entire production period (Molenaar et al. 2008). Keeping in line with the recent trends in the poultry industry, chick’s early nutritional management post hatching becomes progressively more important to success. In this context, feeding of the hatchling required to be designed for promotion of initial growth and gastrointestinal tract (GIT) functionality and stimulation of its health and immune functions. The intestinal epithelial barrier is a key factor that orchestrates gut homeostasis *via* offering communication between the microbiota and underlying intestinal immune cells (Okumura and Takeda 2017; Ibrahim et al., 2023). The cells constructing this layer secrete many antimicrobial proteins and mediators of pro-inflammatory signaling like chemokines, cytokines, and reactive oxygen species (ROS) (Vijay-Kumar et al. 2014). Physiological stress is necessary to a certain degree however, exaggerated physiological inflammation and oxidative stress occasionally, can distributed intestinal epithelial barrier. The intestinal barrier is guarded by tight junction proteins (TJPs), unique proteins, comprising occludin, claudins-1 and JAM which are crucial for creating tight physical barrier among the intestinal epithelial cells and strictly governs their permeability (Nasu et al. 2013). Moreover, TJPs protect the gut from enteric pathogens invasion and prevents the proinflammatory molecules leakage to the circulatory system (Ulluwishewa et al. 2011).

Excessive oxidative stress in the farm environment and physiological inflammation in the GIT can elicit disruption of the intestinal epithelial barrier functions triggering subsequent pathogen invasion that contributes for excessive intestinal inflammatory process hindering birds productivity (Sundaram et al. 2022). Coccidiosis is curiously linked to the modernization and advancement of poultry production with excessive annual global morbidity and mortality losses (Kadykalo et al. 2018). Such parasites exhibit remarkable species-specific sites of development and foci of pathology within the intestinal tract like *Eimeria acervulina*, *E. maxima*, and *E. tenella* are the most commonly observed species in commercial systems of broiler chickens production (Shirley et al. 2005). Coccidiosis impacts transcellular translocation *via* damaging the barrier between enterocytes besides paracellular translocation by impairing the tight junctions between intestinal cells (Teng et al. 2021). In spite of the treatment and prevention capability of chemotherapeutic agents against coccidiosis, potential higher public concerns arise from residues of these drugs in poultry products and the emergence of drug-resistant pathogens have restricted their use (Abdelrahman et al. 2014; Foroutankhah et al. 2019; Hussain et al. 2022; El-Ghareeb et al. 2023; Yaqub et al. 2023). Hence, there is an increasing necessity for novel natural alternative additives to improve broilers performance and disease resistance (Landy et al. 2020).

Recently, natural immunomodulatory nutrients as the omega-3 polyunsaturated fatty (PUFAs) can support bone health, antioxidant response, the intestinal equilibrium maintenance even under stress conditions *via* augmenting epithelial regeneration and proliferation, exerting a potential anti-inflammatory, antimicrobial properties and augmenting intestinal barrier (Rustan and Drevon 2001; Tompkins et al. 2022). Omega −3 poly unsaturated fatty acids (PUFAs) not only cease inflammation but also cleans inflammatory remains and promotes antimicrobial defense for gut homeostasis (Sundaram et al. 2022). As long, n-3 PUFAs are considered important due to the lacked ability of avian species to insert a double bond beyond Δ − 9 carbon due to Δ-12 and − 15 desaturases deficiency, these essential fatty acids must be provided through the diet especially in post hatching period (Thanabalan and Kiarie 2021).

It was found that dietary supplementation of omega-3 PUFAs significantly improved the feed conversion ratio, body weight gain, immune response and anti-oxidative properties of broilers during the entire rearing period (Abdulla et al. 2017; Donaldson et al. 2017)

On the other hand, glutamine (Glut) is significantly assumed as a conditional indispensable amino acid uniquely in challenging periods, is an principal energy fuel for immune cells and commonly demonstrates on the list of ‘immunonutrients’ and exerts antioxidant functions (Bai et al. 2019, p. 6. Particularly for gut, glut has been reported to support gut development and protect the intestinal epithelium in animal models, especially during stresses (Li et al. 2004; Sallam et al. 2023). The mechanism of its protective effects may be a result of improved tight junction proteins (TJP) expression in the small intestine and reduced intestinal permeability during the periods of stresses (Wang et al. 2015). The impact of dietary glutamine supplementation on broilers’ better growth performance was lately documented (Abdulkarimi et al. 2019; Oxford and Selvaraj 2019) while its effect when used in early feeding of life and how it impacts the entire rearing cycle especially at the molecular levels didn’t fully investigated until now. Recently, it has been found that blends of feed additives, especially ones of unique properties, give better outcomes on broilers’ performance and disease resistance than employing single one in their feeding (Kishawy et al. 2022). The importance of dietary supplementation of omega-3 and Glut post hatching as promising insights toward effective broilers’ productive cycle has not been recognized until now. In this scenario, the current study, for the first time, designed to underline the mechanisms considering the synergistic outcomes of Glut and omega-3 for combating coccidian infection as well as promoting growth performance when administered in early nutrition regimen. Herein combined impact of Glut and different levels of omega-3 was assessed *via* investigating growth performance, tissues parameters and molecular aspects controlling muscle building, antioxidant status and intestinal barrier immunity of broiler chickens during experimental infection with Eimeria ssp.

## 2. Materials and methods

### 2.1. Ethical statement

The birds’ arrival guidelines, husbandry practices and ethics were done in according to the guidelines of Institutional Animal Care and Use Committee of the Faculty of Veterinary Medicine at Zagazig University (ZU-IACUC/2/F/260/2023).

### 2.2. Materials

L-glutamine powder was purchased from Sigma Aldrich CO. 2023 Merck KGaA, Darmstadt, Germany (product No: G8540; which consisted of 99–100% pure L-Glutamic acid 5-amide from non-animal source). Omega-3 product was obtained from Natrol LLC 21411 Prairie Street Chatsworth USA.

### 2.3. Experimental chicks, design, and formulated diet

The present study was carried out on a total of 600 one-day-old male broiler chickens Cobb 500 that were gained from a commercial hatchery (average body weight of 44.50 ± 0.25 g) and were randomly assigned into six dietary treatments (five replicates/group with 20 chicks per replicate). During random distribution to represented pens, all chicks were individually weighed, and their leg was banded. All birds were reared under the same controlled managemental housing environmental, and hygienic conditions for 35 days according to Vantress Cobb 500 broilers management guidelines (Cobb 2013). The temperature was adjusted to 30 ± 1 °C for the first 3 days and reached 25◦C at the end of the experimental period and humidity was kept around 60% through the entire experimental period. The lighting regime was 24 h from days 1 to 5 and then 23 h lighting was employed until the end of the experiment. Cobb broilers were received three stages experimental diets: starter (crumb form 1 to 10 d), grower (pellet form 11 to 22 d) and finisher (pellet form 23 to 35 d) diets and formulated to adjust the Cobb 500 broilers nutrient requirements consistent with the nutritional specification of Vantress Cobb 500 broilers which presented in Table 1. All Cobb broilers’ chicks had free access to water and feed. The proximate nutrients’ analysis of the feed ingredients and diets (dry matter, crude protein, ether extract, crude fiber, and Ash) was accomplished in agreement with the method of the Association of Official Agricultural Chemists (AOAC 2012). The six experimental groups were designed as following: the first group: Control, un supplemented); birds fed un supplemented basal diet and unchallenged and the other five groups: (challenged groups); whereas the birds in second group were received un-supplemented control diet (negative control, NC), birds in third group were received L-glutamine supplemented diet (Glut), birds in the fourth, fifth and six groups received L-glutamine supplemented diet with omega-3 by three levels (Glut-omega3I, Glut-omega3II and Glut-omega3III). L-glutamine was supplemented at the level of 1.5% and omega-3 was supplemented at the levels of 0.25, 0.5 and 1%, respectively over the starter period (1–10 days).

**Table 1.** Feed ingredients and chemical analysis of the basal diet (as dry matter).

Ingredients g/kg	Starter (0–10day)	Grower (11–22 day)	Finisher (23–35 day)
Yellow corn grain	59.65	63.40	63.00
Soybean meal, 47.5%	34.50	26.10	23.50
Corn gluten, 60%	1.00	3.50	3.50
Wheat bran	–	2.55	3.25
Soybean oil	–	–	2.60
Calcium carbonate	0.80	1.00	1.00
Dicalciumphosphate	2.30	1.70	1.50
NaCl	0.50	0.50	0.50
Premix*	0.50	0.50	0.50
DL-methionine, 98%	0.20	0.20	0.15
Lysine, Hcl, 78%	0.20	0.20	0.15
Anti-mycotoxin	0.10	0.10	0.10
Sodium bicarbonate (Na_2_Co_3_)	0.25	0.25	0.25
Analyzed chemical composition			
ME, Kcal/Kg**	2906.79	2950.41	3106.38
CP, %	21.99	20.13	18.87
EE, %	2.59	2.72	5.23
CF, %	2.67	2.77	2.74
Ca, %	1.02	0.92	0.87
Available P, %	0.56	0.44	0.40
Lysine, %	1.39	1.16	1.06
Methionine, %	0.55	0.54	0.48
Methionine + cysteine %	0.94	0.91	0.82
Threonine, %	0.88	0.82	0.75

* Vitamin and mineral premix supplied per kg of diet as follow: Vitamin A, 12 500 IU; Vitamin D3, 2300 IU; Vitamin E, 30 IU; Vitamin K3, 6.00 mg; Vitamin B1, 3.85 mg; Vitamin B2, 6.62 mg; Vit5amin B6, 1.6 g; Pantothenic acid, 20 mg; Vitamin B12, 0.5 mg; Niacin, 40 mg; Folic acid, 1.5 mg; Biotin, 0.7 mg; Fe, 55 mg; Mn, 65 mg; Cu, 7 mg; I, 0.9 mg; Co, 1.2 mg; Se, 0.30 mg; Zn, 55 mg; Choline chloride, 600 mg.

** ME calculated according to National Research Council (NRC 1994).

** ME, metabolic energy; CP, crude protein; EE, ether extract; CF, crude fiber; Ca, calcium; P, Phosphorus.

### 2.4. Mixed Eimeria spp challenge

Oocysts were preserved *via* 2.5% potassium dichromate solution, propagated according to technique of Long et al. (1976), then washed numerous times with pure water for removal of potassium dichromate. The degree of sporulated coccidian oocysts per mL was checked by microscope applying a McMaster counting chamber. On day 14 of age, all birds in challenged groups were experimentally infected with mixed species of *Eimeria. via* gavaging a suspension of sporulated oocysts (2 mL) from *E. maxima* (7.0 x 10^3^), and *E. acervulina* (3.5 x 10^4^) and *E. tenella* (5.0 x 10^3^) into the crop *via* a syringe adapted with plastic cannula. The reference stocks of *Eimeria spp* were provided by the Department of Parasitology at the Veterinary Medicine Faculty of Zagazig University, Zagazig. The number of coccidian oocysts per mL was checked prior to gavaging the birds *via* microscope *via* a McMaster counting chamber.

### 2.5. Growth performance assessment

The body weight (**BW**) of the broilers was determined at the end of each feeding stage, and average feed intake (**FI**) was calculated every day from 1 to 35 then the body weight gain (BWG) and feed conversion ratio (FCR) were calculated accordingly as formerly stated by Kishawy et al. (2022) for the whole experimental period. Daily mortality was observed then calculated for each experimental replicate at the end of the experimental period and expressed as a percentage of total number of birds.

### 2.6. Sampling

All sampling procedures and techniques were done under standard hygienic conditions which agreed with faculty of Veterinary Medicine, Zagazig University, Zagazig, Egypt.

#### 2.6.1. Blood sampling

Blood samples at 10 and 20 dpi were aseptically obtained from the wing vein *via* sterile syringe (five bird/group) with anticoagulant EDTA tubes for hematological analysis and without anticoagulant to separate serum through centrifugation for 15 min at 3000 × g for further biochemical and immunological analysis.

#### 2.6.2. Tissues and intestinal content collection

After blood collection, the same birds were euthanized, and slaughtered *via* cervical dislocation for immediate intestinal tissues were collected aseptically for cytokines, immune and antioxidant analysis at 10 and 20 dpi. Intestinal samples (cut to 1-cm longitudinal sections) were weighed and homogenized in 10 mL potassium phosphate buffers with pH 6.0, hexadecyl trimethyl ammonium bromide, and ethylene acetic acid. In the next step, the homogenates were centrifuged for 20 min at 2000 rpm for collection of supernatants until further analysis. Jejunal samples (at 10 and 20 dpi) were rapidly cut out and rinsed by ice-cold phosphate-buffered saline and breast muscle tissues approximately one g (at d 12 of age pre-infection) were collected and, then tissues samples were immediately preserved in liquid nitrogen for later isolation of RNA and gene expression by real-time PCR.

Cecal contents were collected, dissolved in NaCl solution (0.9%; 1:1, *v*/v) and centrifuged for 1 min at 10000 × g. In the next step, the secreted immunoglobulin A (sIgA) concentrations in the supernatant were used to determine sIgA.

Bone sampling: the tibia was removed from the thigh muscles of each slaughtered bird, their cartilages also separated. Then weighed and dried in hot air oven and stored on room temperature tell further calcium and phosphorus analysis.

### 2.7. Tibial mineral content

For analysis of calcium and phosphorus content of tibia firstly, it is combusted in muffle furnace at 550 °C for 48 h. Calcium and phosphorus quantity in tibia ash were analyzed using an atomic absorption according to (Abdel-Raheem et al., 2023) and Perkin-Elmer (1968). In brief, 2 mLof perchloric acid and 3 mL of nitric acid was added to each I g of sample ash. The mixture incubated overnight at 25 °C and then incubated in a hot water bath for 3 h at 72 °C, then filtrated using Whatman paper; the filtrate further completed with deionized water to 25 cm. Aliquot part of the sample’s filtrates were used to examine the concentration of calcium and phosphorus by an atomic absorption spectrophotometer (model 210 VGP, Buck Scientific USD) using an oxidizing air acetylene flame.

### 2.8. Serum biochemical and immune assay and blood hematology

Serum biochemical profile including total protein, uric acid, creatinine, alanine transaminase (ALT) and aspartate transaminase (AST) was assessed by semiautomatic clinical chemistry analyzer using Bayer diagnostics reagents strips following the manufacturer’s direction. The activities of lysozyme were estimated *via* a turbidimetric assay (Shah et al. 2009) by lysozyme activities kit of Sigma-Aldrich (LY0100, Chemie GmbH Eschenstrasse, Germany). Red blood cells (RBCs) were evaluated *via* a Neubauer hemocytometer counting chamber (Sigma, Germany) and blood hemoglobin (Hb) were examined by the procedure of cyanomethemoglobin colorimeteric.

### 2.9. Assessment of intestinal myeloperoxidase, nitric oxide and nitric oxide synthase

Intestinal myeloperoxidase (MPO), nitric oxide (NO) and nitric oxide synthase (NOS) were estimated calorimetrically using assay kits from Sigma-Aldrich (MAK068-1KT MAK454 and MAK532Chemie GmbH Eschenstrasse, Germany).

### 2.10. Enzyme-linked immunosorbent assay (ELISA) for intestinal inflammatory cytokines and sIgA levels in intestinal contents

For assessing the levels of IL-6, TNF-α, and IL-10, the collected supernatants of intestinal tissues from different treatment groups were examined for cytokine secretion utilizing IL-6 ELISA Kit (ab273258), IL-10 ELISA Kit (ab273259) from abcam co. Cambridge Biomedical Campus Cambridge, UK and TNF-α ELISA Kit (E-EL-Ch0215) from Elabscience CO. Houston, Texas, 77079, USA.

The levels of sIgA in cecal content were examined *via* the available ELISA kit (Sigma, St. Louis, MO, USA) according to the production rules. In brief, 96 wells coated with identifiable antibody were incubated at room temperature. After that, diluted samples were added to labeled wells with later secondary antibodies and ortho-phenylenediamine addition. After an incubation period, H_2_SO_4_ was used for stopping the reaction and the absorbance was established at 492 nm.

### 2.11. Assessment of oxidative and antioxidants biomarkers in intestinal tissues

The intestinal tissue homogenate samples were used for assessing the oxidative and antioxidant biomarkers. Total antioxidant capacity (T-AOC) was measured *via* a commercial assay kit (Sigma-Aldrich, MAK187). Glutathione peroxidase (GSH-Px) and superoxide dismutase (SOD) were evaluated by a Sigma Assay Kits (19160, and 219265, respectively)

Hydrogen peroxide (H_2_O_2_) levels were determined in line with Loreto and Velikova (2001) and their concentrations were expressed as μmoL/g of tissue. The measurement of lipid oxidation were defined by the thiobarbituric acid-reactive assay (MDA) value as stated by Ahn et al. (1999) and the values of MDA were expressed as nmol/g tissue. Also, reactive oxygen species (ROS) was measured as stated by the procedure of LeBel et al. (1992).

### 2.12. Assessment of anti-coccidial markers

For counting of fecal oocytes shedding of mixed *Eimeria spp,* the fecal samples from each respective replicas were collected at d 7, 14 and 21 post-infections (dpi) and weighed and homogenized daily. One g of each freshly collected fecal samples was diluted up to 10-fold with distilled water and then re-diluted with 1:10 of saturated saline solution. The number of oocytes/g of faces was counted along with (Christaki et al. 2004).

For evaluating the intestinal lesion score, five birds/group at d 7 (dpi) were used for assessment of intestinal lesion score. Immediately the intestine was plugged out from each bird and differentiated into duodenum, jejunum, ileum and cecum then opened. The lesion scores in intestinal segment were carried out as following: Zero (normal intestinal segment with no gross lesions), one (denoted small distributed petechiae), two (denoted abundant petechiae), 3 (denoted widespread hemorrhage), and 4 (denoted pervasive hemorrhage with dark pigmentation of the intestinal segment (Johnson and Reid 1970).

### 2.13. Genes expression by quantitative real time PCR (qRT-PCR)

Breast meat tissues samples were collected at the end of the stater period for investigating the expression of genes encoding myogenic regulatory factors [myogenin (MyoG), myostatin (MSTN), insulin-like growth factor 1 (IGF-1), mammalian target of rapamycin (mTOR), and myoblast determination protein 1 (MyoD)]. Intestinal tissues were collected at 10 and 20 dpi for examining the expression levels of genes encoding tight junction and inflammatory genes [ß-defensin 1, Cathelicidin-2, mucin-2 (MUC-2), occluding and junctional adhesion molecule (JAM)]. The isolation of total RNA was done exploiting QIAamp RNeasy Mini kit (Qiagen, Germany) and RNA quantification was accomplished at 260 nm using spectrophotometer. The assay of one-step RT-qPCR was established on the Stratagene MX3005P real time PCR employing a QuantiTect SYBR Green RT-PCR Kit (Qiagen, Germany). The variance of each PCR amplification assay was justified by testing of last melting curve. All measured samples of PCR were occupied in triplicate. The various transcripts levels were then adapted *via* glyceraldehyde 3-phosphate dehydrogenase (GAPDH) as an internal control. The primer sequences engaged in RT-qPCR assay are illustrated in Table 2. The results concerning relative mRNA expression of studied genes were estimated *via* 2^−ΔΔCt^ approach (Livak and Schmittgen 2001).

**Table 2.** Primers’ sequences employed for reverse transcription-quantitative polymerase chain reaction assay.

Specificity/target gene	Primer sequence (5’-3’)	Accession no.
*MYOG*	F-CCT TTC CCA CTC CTC TCC AAA R-GAC CTT GGT CGA AGA GCA ACT-	NM_204184
*MYOD1*	F-GAC GGC ATG ATG GAG TAC AG- R-AGC TTC AGC TGG AGG CAG TA	NM_204214
*MSTN*	F-GCTCAAACAGCCTGAATCCAAT R-ACATCGGGATTCCGTTGAGT	KF721281
*mTOR*	F-AAAGCAGCTCTTCCACCAAA R-TGGCTCGTGCCAACATACTA	XM_417614.8
*IGF-1*	F-TCGCATCTCTTCTATCTGGCCCTGT R-GCAGTACATCTCCAGCCTCCTCAGA	NM_001004384
*JAM-2*	F-AGACAGGAACAGGCAGTGC R-TCCAATCCCATTTGAGGCTA	XM_046907882
*MUC-2*	F-ATTGAAGCCAGCAATGGTGT R-TTGTTGGCCTTGTCATCAAA	XM_040673075
*OCLN*	F-ACGGCAGCACCTACCTCAA R-GGCGAAGAAGCAGATGAG	NM_205128.1
*AVBD1*	F-GATCCTCCCAGGCTCTAGGAAG R-GCCCCATATTCTTTTGC	NM_204993
*CATH2*	F: CCGGGCGTCGATCTGA R: GGTGCACTCTGTCTCCATGATG	NM_001024830.2
*GAPDH*	F: GGTGGTGCTAAGCGTGTTA R: CCCTCCACAATGCCAA	NM205518

Myogenin *(MYOG),* myogenic differentiation *1 (MYOD1),* myostatin *(MSTN),* mechanistic target of rapamycin *(mTOR),* insulin like growth factor-1 *(IGF-1),* Junction adhesion molecule-2 *(JAM-2), mucin-2 (MUC-2),* occludin *(OCLN),* Avian β-defensin 1 *(AvBD1),* Cathelicidin-2 *(CATH2)* and glyceraldahyde-3-phosphate dehydrogenase *(GAPDH)*.

### 2.14. Statistical analysis

The analysis of collected data was done Statistically using general linear method (GLM) of SPSS and the replicate represented an experimental unit. Data for each pen was calculated individually, with a total number of 5 replicates within the group and a total of 30 replicates for all six groups in the experiment. The homogeneity among treatment groups was anticipated *via* Levene’s test and normality was measured by Shapiro–Wilk’s test and There was no significant difference among pens within the same group. Overall Performance data were analyzed using GLM (general linear model) two-way analysis of variance, using PASW statistics 18 (SPSS Inc., Chicago, IL, USA) to clarify the effect of different supplemental feed additives at during varying stages of growth and their interaction. Orthogonal polynomial contrasts were used to detect linear and quadratic effect of glutamine and omega-3 power levels (Ghazaghi et al. 2024). Dunnett’s test was used to investigate the differences between control and experimental groups that received glutamine and omega-3. Furthermore, Tukey’s test was done to differentiate the mean values as the deviations were significant. Data difference was presented as standard error of the mean (SEM) and the statistical implication was modified at *p* value less than 0.05. All graphs were generated by GraphPad Prism software Version 8.

## 3. Results

### 3.1. Growth performance indices

The effect of supplementing starter ration of broiler chickens with glut and varying levels of omega-3 fatty acids on growth performance is illustrated in Tables 3 and 4. The presented data were examined *via* the variance test analysis (ANOVA), further the normality and equal variance were determined to meet the assumptions of this statistical test. At the end of starter period (10 days), the BW, BWG and FCR had significantly improved (*p* < 0.05) in all groups supplemented with glut and this improvement was exaggerated in groups fed glut and omega-3 especially the higher level of omega-3 (0.5 and 1%) compared to control and NC groups while the feed intake was non-significant (*p* > 0.05) among all groups. At the end of grower period (22 day), the BW, BWG and FCR of experimental groups supplemented with glut only or glute in combination with omega-3 had increased significantly (*p* < 0.05) in dose dependent manner when compared to NC group however, all these groups still significantly (*p* < 0.05) lower than control. Moreover, the feed intake in grower period recorded a significant (*p* < 0.05) decrease in group fed supplemental Glut + Omega-3II and Glut + Omega-3III groups compared with other groups as the highest value recorded in control group. BW and BWG at finisher period (35 days) were enhanced significantly (*p* < 0.05) in groups fed Glut + Omega-3 especially with 1% omega-3 when compared with challenged non supplemented group (NC). At finisher period, FCR was enhanced in groups fed Glut + Omega-3II and Glut + Omega-3III groups followed by group fed Glut alone or Glut + Omega-3I groups compered to NC group. Groups Glut + Omega-3II or Glut + Omega-3III had showed a significant higher (*p* < 0.05) FI than other experimental groups at finisher period. Concerning the overall feeding period (0–35 days) BW and BWG were linearly and quadratically (*p* < 0.05) increased in challenged groups fed glut or Glut + Omega-3 groups and the improvement correlated to the dose of omega-3 when compared with challenged non supplemented group (NC). Moreover, FCR was restored in group received Glut + Omega-3 III as control group through the entire rearing period. All Meanwhile FI showed no significant differences (*p* > 0.05) between all experimental groups. The statistical analysis showed that the time had significant (*p* < 0.001) impact on overall performance traits among all experimental groups. Moreover, the interaction between time and treatments on total FI, BW, BWG and overall FCR was significant (*p* < 0.001). However, a comparison of the control with the omega-3 supplemented groups showed that omega-3 significantly changed the BW, BWG and FCR while FI showed no significant changes.

**Table 3.** Impact of newly formulated post-hatching diet reinforced with glutamine and different levels of omega-3 on starter, grower and finisher growth performance (0–35 days) of broiler chickens.

Parameters	Control	NC	Glut	Glut + Omega-3I	Glut + Omega-3II	Glut + Omega-3III	*p* Value	SEM
Starter period (1–10 day)								
Initial BW(g/bird)	44.60	44.73	44.40	44.26	44.72	44.61	0.361	0.07
BW (g/bird)	262.10^c^	262.32^c^	310.60^b^	311.54^b^	327.20^a^	329.06^a^	<0.001	5.20
BWG (g/bird)	217.50^c^	217.59^c^	266.20^b^	267.28^b^	282.48^a^	284.45^a^	<0.001	5.21
FI (g/bird)	312.98	313.00	313.14	312.96	313.04	313.00	0.998	0.11
FCR	1.44^a^	1.44^a^	1.17^b^	1.17^b^	1.11^c^	1.10^c^	<0.001	0.03
Grower period (11–22 day)								
BW (g/bird)	1202.80^a^	916.00^f^	985.80^e^	1044.40^d^	1087.40^c^	1183.40^b^	<0.001	18.96
BWG (g/bird)	940.70^a^	653.68^f^	675.20^e^	732.86^d^	760.20^c^	854.34^b^	<0.001	18.65
FI (g/bird)	1181.80^a^	1275.40^b^	1257.40^b^	1250.60^c^	1199.60^d^	1178.00^d^	<0.001	7.22
FCR	1.26^f^	1.95^a^	1.86^b^	1.71^c^	1.58^d^	1.38^e^	<0.001	0.05
Finisher period (23–35 day)								
BW (g/bird)	2492.00^a^	1900.00^f^	2076.00^e^	2118.40^d^	2264.20^c^	2368.00^b^	<0.001	37.49
BWG (g/bird)	1289.20^a^	984.00^d^	1090.20^c^	1074.00^d^	1176.80^b^	1184.60^b^	<0.001	19.94
FI (g/bird)	2170.00^a^	1976.00^b^	2076.00^ab^	2065.00^b^	2108.00^a^	2096.00^a^	<0.001	22.23
FCR	1.69^d^	2.01^a^	1.90^b^	1.92^b^	1.79^c^	1.77^c^	<0.001	0.03

BW = body weight; BWG = body weight gain; FI = feed intake; FCR = feed conversion ratio. ^a–f^Means within a row carrying different superscript letters denote significant differences (*p* < 0.05). Control: birds fed basal diet; NC (negative control): birds fed basal diet and challenged with *Eimeria spp* at d 14 of age; GLUT: birds fed basal diet supplemented with glutamine at the level of 1.5% and challenged with *Eimeria spp* at d 14 of age; GLUT + omega 3 I, II and III: birds fed basal diet supplemented with glutamine at the level of 1.5% with omega 3 at levels of 0.25, 0.5 and 1% and challenged with *Eimeria spp* at d 14 of age.

**Table 4.** Impact of newly formulated post-hatching diet reinforced with glutamine and different levels of omega-3 on overall growth performance (0–35 days) of broiler chickens.

	Overall growth performance (0–35 day)			
	Final BW (g/bird)	Total BWG (g/bird)	Total FI (g/bird)	Overall FCR
Groups				
Control	2492.00	2447.40	3664.78	1.50
NC	1900.00	1855.27	3564.40	1.92
Glut	2076.00	2031.60	3646.54	1.79
Glut + Omega-3I	2118.40	2074.14	3628.56	1.75
Glut + Omega-3II	2264.20	2219.48	3620.64	1.63
Glut + Omega-3III	2368.00	2323.39	3587.00	1.54
*SEM*	37.49	37.49	12.57	0.03
Probabilities				
Treatment	< 0.001	< 0.001	0.006	< 0.001
Time	< 0.001	< 0.001	< 0.001	< 0.001
Treatment × Time	< 0.001	< 0.001	< 0.001	< 0.001
Control vs. treatments	< 0.001	< 0.001	0.054	< 0.001
Linear response	0.001	0.001	0.139	< 0.001
Quadratic response	< 0.001	< 0.001	0.172	< 0.001

BW = body weight; BWG = body weight gain; FI = feed intake; FCR = feed conversion ratio. ^a–f^Means within a row carrying different superscript letters denote significant differences (*p* < 0.05). Control: birds fed basal diet; NC (negative control): birds fed basal diet and challenged with *Eimeria spp* at d 14 of age; GLUT: birds fed basal diet supplemented with glutamine at the level of 1.5% and challenged with *Eimeria spp* at d 14 of age; GLUT + omega 3 I, II and III: birds fed basal diet supplemented with glutamine at the level of 1.5% with omega 3 at levels of 0.25, 0.5 and 1% and challenged with *Eimeria spp* at d 14 of age.

### 3.2. Breast muscle myogenic regulatory genes

**Figure 1.**
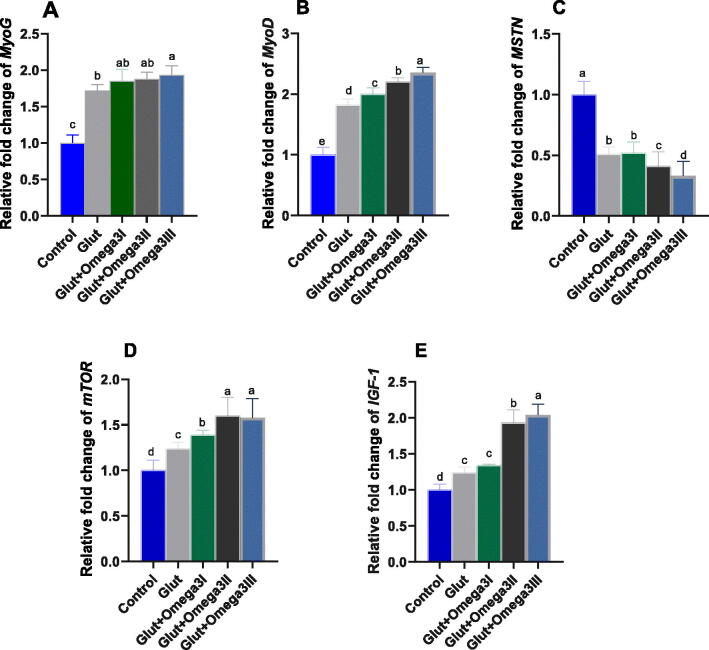
Impact of newly formulated post-hatching diet reinforced with glutamine and different levels of omega-3 on genes expression of myogenesis: myogenin (MyoG, a); myogenic differentiation 1 (MyoD, b); myostatin (MSTN, c); mammalian target of rapamycin (mTOR, d); insulin-like growth factor 1 (IGF-1, e) of broiler chickens before challenge. Control: birds fed basal diet and unchallenged; GLUT: birds fed basal diet supplemented with glutamine at the level of 1.5%; GLUT + omega 3 I, II and III: birds fed basal diet supplemented with glutamine at the level of 1.5% with omega 3 at levels of 0.25, 0.5 and 1% and challenged with *Eimeria spp* at d 14 of age. ^a–e^Mean values with different in the columns are significantly dissimilar at *p* < 0.05.

The expression levels of genes encoding myogenic regulatory factors in broilers breast muscle at the end of stater period in response to glut alone or with different levels of omega-3 are presented in Figure 1. Gene expression of MyoG, MyoD, and IGF-1 were significantly (P < 0.05) up-regulated in groups supplemented with glut or Glut + Omega-3 in level dependent way compared to control group. Concerning to mTOR gene, the highest (P < 0.05) expression level was observed in Glut + Omega-3II and Glut + Omega-3III supplemented groups. In contra, the transcriptional level of MSTN gene showed significant (P < 0.05) down-regulation in groups fed glut with or without different levels of omega-3 in a proportional manner.

### 3.3. Bone calcium and phosphorus content

As illustrated in Table 5. The contrast analysis revealed that Broiler chicken fed Glut + Omega-3III showed a linear and quadratic increase in Tibia bones’ dry matter, ash, calcium and phosphorus percent than another experimental group. Also, control experimental group demonstrated significant higher (P < 0.05) DM%, ash %, calcium and phosphorus % than NC group. Furthermore, no significant differences (P >0.05) recorded in tibia DM%, ash %, calcium and phosphorus % between control, Glute and Glut + Omega-3I groups.

**Table 5.** Impact of newly formulated post-hatching diet reinforced with glutamine and different levels of omega-3 on tibial mineralization, calcium and phosphorus percent of broiler chickens.

									Probabilities		
Parameters	Control	NC	Glut	Glut + Omega-3I	Glut + Omega-3II	Glut + Omega-3III	SEM	*p* Value	Linear	Quadratic	Control vs. treatments
Tibia bone DM %	94.11^c^	93.20^d^	94.10^c^	94.18^c^	94.75^b^	95.10^a^	0.27	<0.001	0.008	0.020	0.038
Tibia ash % of DM	54.37^c^	53.22^d^	54.40^c^	54.43^c^	54.87^b^	55.26^a^	0.22	<0.001	0.004	0.030	0.032
Calcium % of ash	38.07^c^	37.18^d^	38.11^c^	38.14^c^	38.50^b^	38.93^a^	0.12	<0.001	<0.001	0.0350	0.042
Phosphorus % of ash	16.51^c^	15.92^d^	16.53^c^	16.61^ac^	17.18^b^	17.66^a^	0.16	<0.001	<0.001	0.020	0.025

ND: non detected, dpi: days post infection. Control: birds fed basal diet; NC (negative control): birds fed basal diet and challenged with *Eimeria spp* at d 14 of age; GLUT: birds fed basal diet supplemented with glutamine at the level of 1.5% and challenged with *Eimeria spp* at d 14 of age; GLUT + omega 3 I, II and III: birds fed basal diet supplemented with glutamine at the level of 1.5% with omega 3 at levels of 0.25, 0.5 and 1% and challenged with *Eimeria spp* at d 14 of age. ^a–d^Mean values with different in the rows are significantly dissimilar at *p* < 0.05.

### 3.4. Intestinal oxidant and antioxidant associated parameters

As displayed in Table 6, significant higher levels of T-AOC and antioxidant enzymes activities’ including SOD and GSH-PX (*p* < 0.05) were noticed with increasing the supplemental level of Omega-3 in intestinal tissue at 10 and 20 dpi. Intestinal SOD and GSH-PX levels in Glut + Omega-3 supplemented groups were decreased (*p* < 0.001) linearly at 10 and 20 dpi while the quadratic effect was not significant at 10 and 20 dpi. The effect of supplemental Glut + Omega-3 showed linear and quadratic decrease in H_2_O_2_ at 10 and 20 dpi while ROS levels were linearly and quadratically reduced at 10 dpi with linear dropped at 20 dpi. A noticeable linear and quadratic decrease in lipid peroxidation biomarkers (MDA) at 10 and 20 dpi was found in groups fed Glut + Omega-3II and Glut + Omega-3III in intestinal tissue. Remarkable decline (*p* < 0.05) in oxidation markers (H_2_O_2_ and ROS) production was detected in intestinal tissues of groups fed Glut + Omega-3II or Glut + Omega-3III at both 10 and 20 dpi.

**Table 6.** Impact of newly formulated post-hatching diet reinforced with glutamine and different levels of omega-3 on antioxidant and oxidative stress related parameters post challenge.

									Probabilities		
Parameters	Control	NC	Glut	Glut + Omega-3I	Glut + Omega-3II	Glut + Omega-3III	SEM	*p* Value	Linear	Quadratic	Control vs. treatments
At 10 days post challenge											
T-AOC, (U/mg of protein)	1.56^e^	1.20^f^	1.75^d^	1.91^c^	2.07^b^	2.26^a^	0.05	0.01	<0.001	0.005	<0.001
MDA, (nmol/g tissue)	12.31^b^	25.72^a^	11.57^b^	9.92^bc^	8.83^c^	8.03^c^	0.98	0.01	<0.001	0.005	<0.001
H_2_O_2_ (µmoL/g tissue)	2.20^b^	4.17^a^	2.30^b^	2.00^bc^	1.65^cd^	1.19^d^	0.66	0.03	<0.001	<0.001	0.001
ROS, (µL/g tissue)	43.40^b^	73.88^a^	41.87^b^	36.91^b^	30.43^c^	28.47^c^	0.54	0.04	<0.001	0.002	<0.001
GSH-PX, (U/mL)	218.88^d^	151.62^e^	256.73^c^	273.09^bc^	262.44^ab^	283.61^a^	1.47	0.04	<0.001	0.559	<0.001
SOD, (U/mL)	92.40^d^	66.01^d^	114.92^c^	127.25^c^	134.34^ab^	141.40^a^	1.05	<0.001	<0.001	0.888	<0.001
At 20 days post challenge											
T-AOC, (U/mg of protein)	1.58^d^	1.27^e^	2.11^c^	2.29^b^	2.39^ab^	2.48^a^	0.04	0.01	<0.001	0.019	<0.001
MDA, (nmol/g tissue)	11.43^b^	27.79^a^	9.73^c^	7.41^d^	6.30^e^	5.27^f^	1.76	0.01	<0.001	0.001	<0.001
H_2_O_2_ (µmoL/g tissue)	1.85^b^	4.43^a^	1.51^c^	1.36^c^	1.25 ^cd^	1.19^d^	0.23	0.03	<0.001	0.002	<0.001
ROS, (µL/g tissue)	42.43^b^	77.00^a^	28.80^c^	27.43^c^	26.23 ^cd^	22.25^d^	0.43	0.04	<0.001	0.636	<0.001
GSH-PX, (U/mL)	228.47^d^	150.40^e^	261.33^c^	277.69^bc^	267.04^b^	295.41^a^	0.52	0.04	<0.001	0.170	<0.001
SOD, (U/mL)	93.87^d^	65.50^e^	126.93^c^	136.15^bc^	143.24^b^	159.60^a^	0.64	<0.001	<0.001	0.978	<0.001

Control: birds fed basal diet; NC (negative control): birds fed basal diet and challenged with *Eimeria spp* at d 14 of age; GLUT: birds fed basal diet supplemented with glutamine at the level of 1.5% and challenged with *Eimeria spp* at d 14 of age; GLUT + omega 3 I, II and III: birds fed basal diet supplemented with glutamine at the level of 1.5% with omega 3 at levels of 0.25, 0.5 and 1% and challenged with *Eimeria spp* at d 14 of age. Malondialdehyde (MDA); total antioxidant capacity (T-AOC); reactive oxygen species (ROS), superoxide dismutase (SOD); Glutathione peroxidase (GPX-SH); hydrogen peroxide (H2O2). ^a–f^Mean values with different letters in the column are significantly dissimilar at *p* < 0.05.

### 3.5. Serum liver and kidney functions and blood hematological parameters

As shown in Table 7, serum ALT levels were significantly elevated (*p* < 0.05) in NC group than control group; meanwhile their levels in Glut + Omega-3II and Glut + Omega-3III supplemented groups were not significantly differ from control group. However, a comparison to control, Glut + Omega-3 supplemented groups did significantly altered serum ALT, AST, uric acid level and RBCs counts. The values of AST at 10 dpi were a significantly evaluated (*p* < 0.05) in NC group while supplementation with Glut + Omega-3III reduced its levels. Regarding serum kidney function tests, uric acid and creatinine levels were significant upraised (*p* < 0.05) in challenged control compared to non-challenged one. In contrast, inclusion of glut with varying levels Omega-3 reduced uric acid and creatinine levels in a dose dependent relation.

**Table 7.** Impact of newly formulated post-hatching diet reinforced with glutamine and different levels of omega-3 on broiler chicken’s serum parameters related to liver and kidney function post challenge.

									Probabilities		
Parameters	Control	NC	Glut	Glut + Omega-3I	Glut + Omega-3II	Glut + Omega-3III	SEM	*p* Value	Linear	Quadratic	Control vs. treatments
At 10 days post challenge											
ALT, U/L	28.26^c^	50.04^a^	43.26^b^	39.81^b^	32.45^c^	30.92^c^	2.04	<0.001	0.001	<0.001	0.118
AST, U/L	45.87^d^	82.13^a^	55.83^b^	55.60^b^	54.03^b^	49.86^c^	0.61	0.02	<0.001	<0.001	0.05
Uric acid, μmol/L	11.12^d^	19.80^a^	16.63^b^	16.10^b^	14.87^bc^	12.48 ^cd^	0.77	<0.001	0.21	<0.001	0.605
Creatinine, mg/dL	0.29^e^	0.81^a^	0.77^ab^	0.71^b^	0.57^c^	0.48^d^	0.07	0.04	0.32	<0.001	<0.001
RBCs (×10^6^/μL)	2.56^a^	1.84^c^	2.11^b^	2.19^b^	2.23^b^	2.44^ab^	0.13	<0.001	<0.001	0.07	0.815
Hb (g/dL)	12.34^a^	8.32^d^	11.65^c^	11.70^c^	11.76^c^	12.11^ab^	0.14	0.02	0.004	0.08	0.516
At 20 days post challenge											
ALT, U/L	28.36^e^	62.04^a^	53.26^b^	46.81^c^	37.45^d^	33.92^d^	2.01	<0.001	<0.001	<0.001	0.029
AST, U/L	42.03^c^	87.13^a^	52.83^b^	52.60^b^	51.03^b^	43.86^c^	0.70	<0.001	<0.001	<0.001	0.314
Uric acid, μmol/L	10.42^d^	18.20^a^	14.44^b^	12.69^c^	10.67^d^	10.12^d^	0.67	<0.001	0.174	0.214	0.335
Creatinine, mg/dL	0.31^c^	0.78^a^	0.48^b^	0.41^b^	0.35^c^	0.32^c^	0.08	<0.001	<0.001	<0.001	0.900
RBCs (×10^6^/μL)	2.59^a^	1.99^c^	2.19^b^	2.21^b^	2.25^b^	2.49^ab^	0.11	0.03	0.001	0.12	0.913
Hb (g/dL)	12.13^a^	8.32^d^	11.70^c^	11.75^c^	11.82^c^	12.10^b^	0.17	0.04	0.042	0.09	0.978

Control: birds fed basal diet; NC (negative control): birds fed basal diet and challenged with *Eimeria spp* at d 14 of age; GLUT: birds fed basal diet supplemented with glutamine at the level of 1.5% and challenged with *Eimeria spp* at d 14 of age; GLUT + omega 3 I, II and III: birds fed basal diet supplemented with glutamine at the level of 1.5% with omega 3 at levels of 0.25, 0.5 and 1% and challenged with *Eimeria spp* at d 14 of age; SEM: standard error of the mean; ALT: alanine transaminase; AST: aspartate transaminase; ^a–e^Mean values with different letters in the column are significantly dissimilar at *p* < 0.05.

The RBCs count and Hb levels at 10 dpi were markedly reduced in NC group while groups received glut and omega-3 III displayed a significant improvement in RBCs count and Hb levels when compared with control. At 20 dpi; liver function indices including ALT and AST showed a significant highest (*p* < 0.05) values in challenged non supplemented group whereas supplementation with glut and different levels of omega-3 alleviated their levels in dose dependent way to be close to normal values. Generally, serum levels of ALT and AST were higher at 20 dpi than corresponding values at 10 dpi. Kidney function tests at 20 dpi as uric acid and creatinine were significant elevated (*p* < 0.05) in NC than control and the supplementation of experimental groups with Glut + Omega-3III significant decreased (*p* < 0.05) their serum levels to normal boundaries. Notably, the count of RBCs and Hb levels at 20 dpi displayed a non-significant quadratic response as they were resorted to normal levels post challenge in group supplemented with glut and omega-3 III.

### 3.6. Evaluation of immune response post mixed Eimeria spp. challenge

The immune response in the serum and intestinal tissue at 10 and 20 dpi was expressed in Table 8. The serum total protein levels at 10 and 20 dpi were quadratically reduced (*p* < 0.01) in NC group than control group meanwhile; groups fed Glut + Omega-3II or Glut + Omega-3III showed a significant (*p* < 0.05) increased levels compared to NC group. On the other hand, serum lysozymes activity at 10 and 20 dpi exhibited the highest levels in challenged groups. Moreover, groups supplemented with Glut + Omega-3II or Glut + Omega-3III groups had a quadratic increased (*p* < 0.05) in lysozymes activity unlike with control group. Concerning the intestinal immune parameters, the intestinal MPO, NO and NOS at 10 and 20 dpi exhibited a significant (*p* < 0.05) higher levels in NC group than control group, at the same time; groups fed glut alone or glut with omega-3 quadratic decreased (*p* < 0.01) the previously mentioned intestinal immune related parameters compared to challenged un-supplemented control. Furthermore, a comparison of the control displayed that Glut + Omega-3 supplemented groups significantly reduced segmental intestinal lesions and fecal oocyte shedding at 7, 14 and 21 dpi.

**Table 8.** Impact of newly formulated post-hatching diet reinforced with glutamine and different levels of omega-3 on immune related parameters post challenge with mixed *Eimeria spp.*

									Probabilities		
Parameters	Control	NC	Glut	Glut + Omega-3I	Glut + Omega-3II	Glut + Omega-3III	*SEM*	*p* Value	Linear	Quadratic	Control vs. treatments
At 10 days post challenge											
TP, (U/mL)	4.06^a^	1.46^e^	2.25^d^	2.69^c^	3.33^b^	3.63^b^	2.07	<0.001	<0.001	<0.001	<0.001
Lysozyme (U/mL)	118.07^e^	207.49^a^	178.79^b^	166.22^c^	160.28^c^	141.62^d^	11.23	<0.001	0.158	<0.001	<0.001
MPO, (U/L)	23.83^e^	55.47^a^	41.53^b^	38.63^c^	35.73^d^	33.19^d^	0.72	<0.001	0.028	<0.001	<0.001
NO, (U/mL)	5.03^d^	14.82^a^	10.23^b^	10.30^b^	9.47^b^	7.66^c^	0.14	0.04	0.310	<0.001	<0.001
NOS, (U/mL)	5.20^d^	9.40^a^	7.63^b^	7.03^bc^	6.83^c^	6.36^c^	0.37	<0.001	0.103	<0.001	0.002
At 20 days post challenge											
TP, (U/mL)	4.00^a^	1.45^e^	2.92^d^	3.58^c^	4.02^b^	4.43^b^	0.09	<0.001	<0.001	<0.001	<0.001
Lysozyme (U/mL)	117.13^d^	185.99^a^	167.41^b^	160.94^b^	142.21^c^	133.47^c^	16.87	<0.001	0.080	<0.001	0.025
MPO, (U/L)	24.22^d^	46.47^a^	32.97^b^	28.17^c^	25.08^d^	24.63^d^	1.21	<0.001	<0.001	<0.001	0.698
NO, (U/mL)	5.20^e^	12.52^a^	10.03^b^	9.25^bc^	8.71^cd^	7.97^d^	0.60	<0.001	0.394	<0.001	<0.001
NOS, (U/mL)	5.06^d^	9.82^a^	6.33^b^	5.55^c^	5.01^d^	4.27^e^	0.94	0.03	<0.001	<0.001	0.018

TP: total protein; MPO: myeloperoxidase; NO: nitric oxide, NOS: nitric oxide synthase. Control: birds fed basal diet; NC (negative control): birds fed basal diet and challenged with *Eimeria spp* at d 14 of age; GLUT: birds fed basal diet supplemented with glutamine at the level of 1.5% and challenged with *Eimeria spp* at d 14 of age; GLUT + omega 3 I, II and III: birds fed basal diet supplemented with glutamine at the level of 1.5% with omega 3 at levels of 0.25, 0.5 and 1% and challenged with *Eimeria spp* at d 14 of age. ^a–e^Mean values with different letters in the column are significantly dissimilar at *p* < 0.05.

### 3.7. Intestinal inflammatory and tight junction gene expression

**Figure 2.**
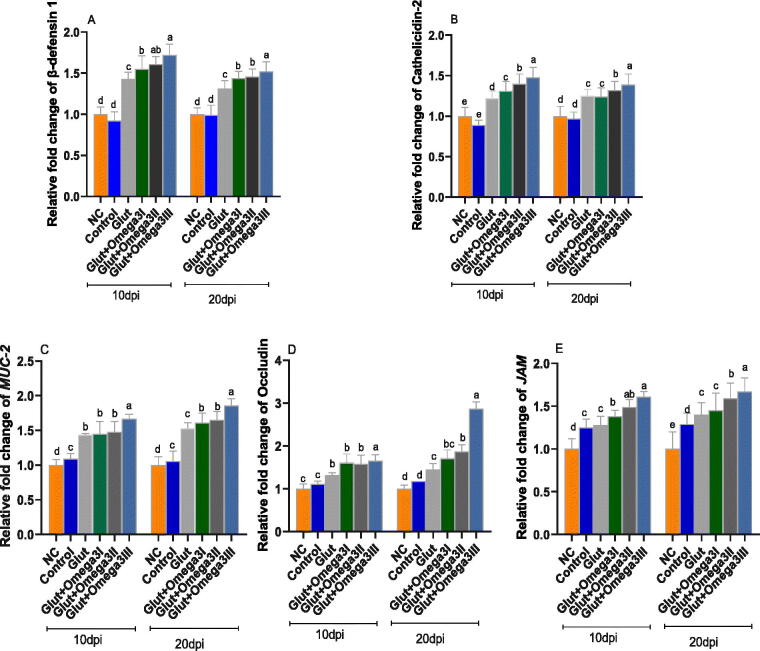
Impact of newly formulated post-hatching diet reinforced with glutamine and different levels of omega-3 on genes expression of intestinal immune defense and tight junction at 10- and 20-days post-infection (dpi). ß-defensin-1 (a); cathelicidins-2 (b); mucin-2 (MUC-2, c); occludin (d); junctional adhesion molecule-2 (JAM, e) of broiler chickens. Control: birds fed basal diet; NC (negative control): birds fed basal diet and challenged with *Eimeria spp* at d 14 of age; GLUT: birds fed basal diet supplemented with glutamine at the level of 1.5% and challenged with *Eimeria spp* at d 14 of age; GLUT + omega 3 I, II and III: birds fed basal diet supplemented with glutamine at the level of 1.5% with omega 3 at levels of 0.25, 0.5 and 1% and challenged with *Eimeria spp* at d 14 of age. ^a–e^Mean values with different in the columns are significantly dissimilar at *p* < 0.05.

As illustrated in Figure 2, the highest expression levels of intestinal inflammatory and tight junction genes including *AvBD1*, Cathelicidin-2 were detected (*p* < 0.05) in Glut + Omega-3III group compared with NC and control un supplemented groups at both 10 and 20 dpi (increased by 1.61 and 1.46, respectively). Furthermore, expression of *MUC-2*, *JAM* and occludin genes was significantly elevated in group fed glut and a dose dependent manner ofomega-3 at 10 and 20 dpi.

### 3.8. Intestinal inflammatory cytokines and sIgA

**Figure 3.**
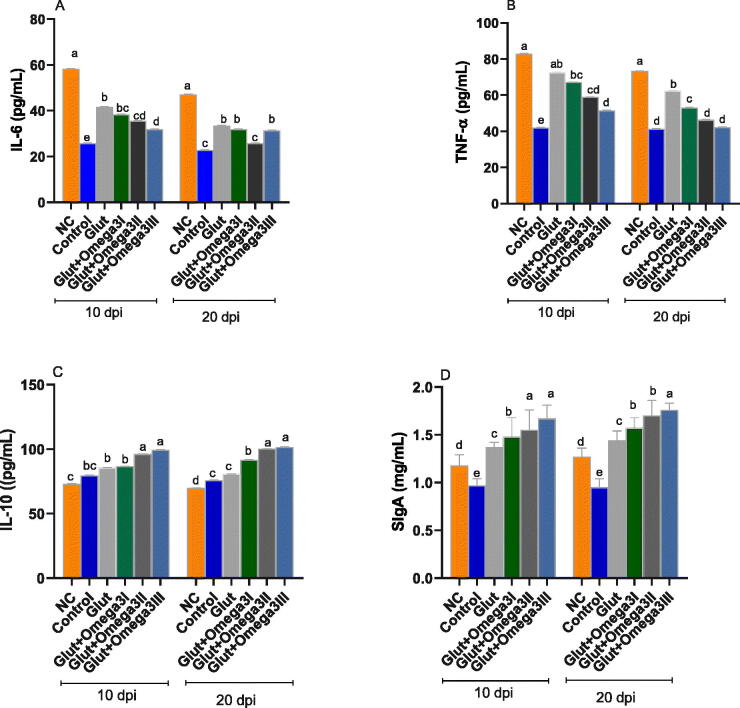
Impact of newly formulated post-hatching diet reinforced with glutamine and different levels of omega-3 on immune related cytokines and SIgA at 10- and 20-days post-infection (dpi). Interleukin-6 (IL-6, a), tumor necrosis factor alpha (TNF-α, B); interleukin-10, (IL-10, C); secretory immunoglobulin A (SIgA, D) of broiler chickens. Control: birds fed basal diet; NC (negative control): birds fed basal diet and challenged with *Eimeria spp* at d 14 of age; GLUT: birds fed basal diet supplemented with glutamine at the level of 1.5% and challenged with *Eimeria spp* at d 14 of age; GLUT + omega 3 I, II and III: birds fed basal diet supplemented with glutamine at the level of 1.5% with omega 3 at levels of 0.25, 0.5 and 1% and challenged with *Eimeria spp* at d 14 of age. ^a–e^ Mean values with different in the columns are significantly dissimilar at *p* < 0.05.

The influence of supplementing broiler starter diets with glut alone or with varying levels of omega-3 on intestinal inflammatory cytokines and **sIgA** at 10 and 20 dpi were shown in Figure 3. The highest levels of inflammatory cytokines as IL-6 and TNF-α at 10 and 20 dpi was detected in NC group, while supplementation with glut and omega-3 significantly (*p* < 0.05) decreased their levels in a dose dependent manner. In contra, the levels of intestinal anti-inflammatory cytokine IL-10 were significantly (*p* < 0.05) decreased in NC group. Meanwhile, groups supplemented with glut and omega-3 significantly (*p* < 0.05) increased its levels in a dose dependent manner at both 10 and 20 dpi. Regarding the intestinal secretory IgA, their highest levels were noted in group fed supplemental Glut + Omega-3III at both 10 and 20 dpi.

### 3.9. Eimeria fecal oocytes count and intestinal lesion score

The influence of supplementing starter diets with glut and different levels of omega-3 on fecal oocytes shedding, intestinal lesion score and mortality % are verified in Table 9. Oocytes of different *Eimeria spp.* were not found in the excreta of control group at all dpi. At 10 and 20 dpi, fecal oocytes count was linearly and quadratically (*p* < 0.01) lowered in groups supplemented with glut alone or Glut + Omega-3I, II and III groups than NC group, while at 21 dpi the significant lowest oocytes count (*p* < 0.05) in feces were detected in Glut + Omega-3II and Glut + Omega-3III supplemented groups. Concerning the intestinal lesion score, NC group exhibited (*p* < 0.05) the highest lesion score in all intestinal segments comprising duodenum, jejunum, ileum and cecum, although the lowest intestinal lesion score was noted in group fed Glut + Omega-3III in all intestinal segments. The total highest mortality % was detected in (*p* < 0.05) in NC group unlike other groups; meanwhile, mortality % was significantly (*p* < 0.05) reduced with increasing the supplemental levels of Omega-3.

**Table 9.** Impact of newly formulated post-hatching diet reinforced with glutamine and different levels of omega-3 on fecal oocytes count, intestinal lesion score and mortality % of broiler chickens post challenge with mixed *Eimeria spp.*

									Probabilities		
Parameters	Control	NC	Glut	Glut + Omega-3I	Glut + Omega-3II	Glut + Omega-3III	*SEM*	*p* Value	Linear	Quadratic	Control vs. treatments
Intestinal lesion score 7 dpi											
Duodenal lesion score	ND	4.00^a^	3.60^a^	3.40^a^	3.00^ab^	2.20 ^b^	0.26	<0.001	0.001	<0.001	<0.001
Jejunal lesion score	ND	3.80^a^	3.60^a^	3.20 ^ab^	2.60^bc^	2.20^c^	0.25	<0.001	0.002	<0.001	<0.001
Ileal lesion score	ND	3.80^a^	3.40^ab^	3.40^ab^	2.80^bc^	2.20^c^	0.25	<0.001	0.001	<0.001	<0.001
Cecal lesion score	ND	3.80^a^	3.60^a^	3.20^a^	2.80^ab^	2.00^b^	0.26	<0.001	0.004	<0.001	<0.001
Fecal oocytes count (×10^3^/g faces)											
7 dpi	ND	357.80^a^	347.60^b^	335.60^c^	326.60^d^	319.20^e^	23.47	<0.001	<0.001	<0.001	<0.001
14 dpi	ND	232.60^a^	218.80^b^	166.00^c^	136.20^d^	123.00^e^	14.22	<0.001	<0.001	<0.001	<0.001
21 dpi	ND	76.20^a^	65.80^b^	53.60^c^	36.00^d^	34.80^d^	4.62	<0.001	<0.001	<0.001	<0.001
Mortality % (1–35 day)											
Mortality %	1.20^f^	37.20^a^	31.40^b^	22.40^c^	15.40^d^	9.00^e^	2.32	<0.001	<0.001	<0.001	<0.001

ND: non detected, dpi: days post infection. Control: birds fed basal diet; NC (negative control): birds fed basal diet and challenged with *Eimeria spp* at d 14 of age; GLUT: birds fed basal diet supplemented with glutamine at the level of 1.5% and challenged with *Eimeria spp* at d 14 of age; GLUT + omega 3 I, II and III: birds fed basal diet supplemented with glutamine at the level of 1.5% with omega 3 at levels of 0.25, 0.5 and 1% and challenged with *Eimeria spp* at d 14 of age. ^a–e^Mean values with different in the rows are significantly dissimilar at *p* < 0.05.

## 4. Discussion

Early feeding programing can positively influence the final weight of broiler chickens (Willemsen et al. 2008). Besides, it can boost the development and functioning of the immune system not only during the early stages of life but also during the whole entire rearing period (Jha et al. 2019).

Coccidiosis has an extensive economic loss, mostly coming from a reduced production performance and prophylaxis and treatment costs, with higher annual global costs for poultry production (Blake et al. 2020). One of the best choice to lessen the usage of antimicrobials controlling coccidiosis that resulted in anticoccidial drugs resistance and public health risk, is the using of dietary interventions that may improve gut health and hence potentially reduce the detrimental impact of coccidiosis on broiler performance (Santos et al. 2022). The pre-starter and starter diets account for good intestinal development, optimal growth and is related to immune function for young birds (Jha et al. 2019). Therefore, providing supportive nutritional aids for birds immediately after hatching is crucial for their future growth and development and protection against pathogens invasion. In this context, applying a combination of nutritional ingredients that can optimize birds’ growth rate, gut health prior to infection, *via* providing antioxidant protection and supporting immune related functions against invasion and multiplication of *Eimeria spp*., is critically needed. In the current study, starter’s growth rate and FCR greatly improved after feeding on formulated diet supplemented with a dietary combination of Glut and omega-3III. These positive impacts were further supported by higher expression of genes accounted for muscle mass development (*MyoG, MyoD* and IGF-1) and down regulation of MSTN gene that negatively regulate of muscle development measured at the end of stater period. Similarly dietary Glut has improved skeletal muscle development and general performance of broilers (Sifa et al. 2018). Also, glutamate stimulated muscle protein synthesis, and whole-body growth and inhibited the intra skeletal muscle proteolysis in chicks (Li et al. 2010; Furukawa et al. 2020). As satellite cell myogenesis and pectoralis growth and development mainly occurs during chick embryogenesis and the early post hatch period (Halevy 2020) thus in the present study, supplementation of Glut immediately post hatching was accounted for an enhanced muscle development and long-term muscle growth.

The better growth performance in all groups supplemented with Glut prior to coccidial infection or even weight gain restoration in these groups post infection came from Glut involvement of in controlling the growth of skeletal muscle and protein synthesis (Dai et al. 2009). Moreover, glutamine supplementation especially at pre-starter and starter diets can trigger broilers’ performance and breast yield (Ribeiro et al. 2015). Accordingly, dietary provision of Glut can enhance birds’ performance, survivability, and well-being during challenge condition (Jazideh et al. 2014). On the other hand, dietary delivery of n-3 PUFAs had a significant positive impact on the formation of bone and its health during early developmental phase (Lau et al. 2013). Post hatching healthy skeletal development parallels to further body growth while the ongoing broilers inability to support their body weight is often resulted from poor bone mineralization and/or muscle inflammation (Tompkins et al. 2022). Omega-3 supplementation can augment the health of musculoskeletal system *via* their positive impact on muscle protein synthesis, anti-inflammatory roles, reduced breakdown of intracellular protein, increased expression of mechanistic target of rapamycin pathway, enhanced mitochondrial function and boosted amino the transportation of amino acids (Therdyothin et al. 2023). There are various theorized mechanisms connecting bone health and n-3 PUFA such as an increase in intestinal absorption of calcium, bone marrow cells modulation and attenuating osteoclastogenesis mediators (Thanabalan and Kiarie 2021). In addition, DHA, EPA is reported to accountable for better muscle protein yield (McGlory et al. 2019). The positive effect of supplementing Glut with higher doses of n-3 PUFAs in the current study may resulted from their boosting functions on bone calcium precipitation formation, and better bone quality (Tompkins et al. 2022). Additionally, omega-3 has a protective function against proinflammatory cytokines that contributes for muscle inflammation and bone loss and weakening (Tong et al. 2020). Furthermore, higher bone calcium content in this study supported the effective role of omega-3 on increasing its absorption and balance in the small intestine that resulted in an augmented calcium availability and consequently its potential incorporation into the bone matrix (Lau et al. 2013).

Newly hatched chicks are facing to a diversity of stressors that promote the stress response, depress immunity and retard bird’s growth rate and well-being. The levels of T-AOC, GSH-Px, SOD and MDA are critical biomarkers in the response of antioxidant system inside the body. In particular, the antioxidant capacity is inversely proportional to MDA levels, that reflects the lipid peroxidation degree and tissues oxidative damage (Luo et al. 2011). The total antioxidant capacity is concerned as the capacity to preserve the peroxidation and antioxidant balance (Kishawy et al. 2023). Antioxidant enzymes comprising GSH-Px, SOD and CAT can alleviate the exaggerated peroxidation products (Ibrahim et al. 2022). Coccidia invasion is among the most important pathogenic microorganisms that primarily stimulates oxidative stress and cause disbalances in antioxidant enzymes and peroxide products expression (Yu et al. 2022). Moreover, excessive production of these free radicals can cause cell cytotoxicity and death and account for disease pathogenesis (Vladimirov 2004). As evidenced in our study, coccidia infection weakened the antioxidant functions of infected broilers (higher contents of MDA, ROS and H_2_O_2_ with reduced activities GPX and SOD in the intestinal tissues) especially at 20 days post infection which in agreement with (Zhang et al. 2017). In contrast the distributed balance in the antioxidant and the pro-oxidative response following infection was greatly restored by supplemental Glut and higher levels of omega 3. In accordance, glutamine supplementation was accounted for higher SOD and GPX activities which concomitant with lower levels of MDA in the serum of broilers (Dong et al. 2009). Also, dietary glutamine may augment tissue antioxidant function in poultry and lower the influences of oxidative stress (Zhu et al. 2011). Antioxidant-enhancing abilities of glutamine could attributed to its involvement in GSH synthesis that inhibited oxidative injury initiated from free radicals and peroxides generation (Cao et al. 1998). Also, glutamine may be attributed to its ability to increase the activity of radical-scavenging enzymes (Zhu et al. 2011). On the other hand, broilers fed on supplemental omega 3-rich oils has shown an enhanced antioxidant response. Similarly, incorporation of fish and linseed oils rich in omega-3 lowered serum levels of MDA and increased glutathione levels (Ibrahim et al. 2018). Additionally, omega-3 probably have antioxidant impacts by suppressing lipid peroxidation (Da Silva et al. 2016), elevating GPX activity and increasing T-AOC due to decreasing of Ros (Iraz et al. 2005). Moreover, n-3 PUFAs have lately been known to depress pro-oxidant activity *via* upregulating cytoprotective antioxidant genes including glutathione peroxidase and heme oxygenase 1 and lowering endogenous ROS levels (Zhang et al. 2014). The functional role of plants derived bioactive compounds in defense against coccidiosis may be associated with their monitoring impact on lipids peroxidation in intestinal cells and reducing ROS and NOS creation and accordingly their dangerous impacts (Abbas et al. 2013). Similarly, findings described by Idris et al. (2017) cleared that addition of antioxidants enriched essential fatty acids can relieve the oxidative stress which produced from *Eimeria spp* invasion. Consistent with our results Tsiouris et al. (2021) stated enhanced antioxidants markers and mitigation of coccidian infection proved post supplementation of dietary antioxidants. In the current study, the dual effects of Glut and omega 3 on alleviating the oxidative stress associated with coccidia infection in broilers can be related to their augmented scavenging activity of free radicals.

The intestinal epithelial barrier is a key factor that orchestrates gut balance *via* creating communication between the microbiota and underlying immune lining cells (Okumura and Takeda 2017). This barrier is guarded by tight junction proteins (TJPs), unique proteins, comprising occludin, claudins-1 and JAM which are crucial for providing integral physical barrier between intestinal mucosal cells and strictly governs its permeability (Nasu et al. 2013). Coccidiosis impairs digestive capacity and absorption and barrier function in coincidence with elevated risks for secondary bacterial infections (Chapman et al. 2016). Herein, TJPs were impaired along with coccidia infection as evinced by downregulation of encoding controlling genes occludine, mucin and *JAM*. Accordingly, *Eimeria* can induce oxidative stress with excessive production of free radical that injuries intestinal barrier and subsequently facilitate its further intestinal invasion (Dos Santos et al. 2020). Moreover, Chen et al. (Chen et al. 2015) has conveyed that higher content of cytokines and subsided occludine can prompt failure of gut barrier and jejunum mucosal inflammation and resulting in prominent elevated endotoxin levels in broilers serum (Chen et al. 2015). Additionally, gene expression of occludine and *JAM-2* was downregulated by mixed *Eimeria spp* challenge at d 6 dpi (Teng et al. 2021). However, dietary supplementation of Glut + omega-3III greatly upregulated these genes which suggested their effective role in strengthen the mucosal barrier and enhancing the intestinal integrity (Verwoolde et al. 2021). In addition, mucin in the gastrointestinal tract considered as the first line of immune protection and the enhancement of its production could be favorable in hindering invasion of pathogenic microbes and their toxins (Murai et al. 2018) Whilst decreasing the MUC-2 expression, regulating mucin secretion, in the challenged supplemented group can be associated with excessive gut inflammation. Furthermore, inflammatory lesions can reduce goblet cells producing mucin, inhibit regeneration of mucosal layer and boost infection, bacterial translocation and further intestine inflammation (Forder et al. 2012). Herein, prophylactic reinforcement of intestinal barrier following to infection was clearly observed ingroup fed on supplemental Glut and/or omega-3 by the level of 1%. Concurrently, glutamine supplementation can increase survival, growth performance and gut-barrier functions in livestock under injury and stress state (Zhong et al. 2012). The effective role of n-3 PUFAs in restoring intestinal barrier integrity through antagonizing inflammation by replacing ω-6 arachidonic acid, pro-inflammatory eicosanoids described to modify the gut microbiota and interrupts the intestinal barrier functions, and yield 100-fold weak pro-inflammatory eicosanoids that accounted for inflammation un-recovery (Calder 2013). Additionally, ω-3 PUFA enhanced the expression of tight junction genes (E-cadherin and ZO-1), and tissue restoration were significantly enhanced with the abundance of goblet cells (Liu et al. 2012). Restoring intestinal barrier functions and subsiding of inflammatory mediators by supplementing glutamine and Omega-3 indicating their protective role against gut injury associated with *Eimeria* infection.

Endogenous host defense peptides (HDPs) have a crucial protective barrier in the intestine, play multiple key physiological functions in the gut mucosa and enhance wound healing (Brogden et al. 2012). Furthermore, boosting these endogenous peptides is considered as a novel strategy for struggling pathogens, and to sustain intestinal health and decrease the use of antibiotic (Wu et al. 2020). Cathelicidins, along with defensins belong to the large group of cationic peptides with an important functions in the immune system in humans and farm animals (Kościuczuk et al. 2012) is orchestrating the innate host defense (Lee et al. 2005).

Cathelicidins are small peptides with antimicrobial effects intermediated through interaction with the cell membrane of intestinal microbes (Steinstraesser et al. 2011). The defensins are antimicrobial peptides enriched with cystine with wide-ranging antimicrobial activities through triggering macrophages cells to reach mucosal tissues by chemokine receptors and furthermore enhance immunity tackling specific pathogens (Wang et al. 2003). Likewise, defensins exert their effect directly on bacterial pathogens and more prominently exert a vital function in the innate immunity assisting in adaptive immune response stimulation and regulation (Guo et al. 2017). In line with the previous-declared facts, expression of β-defensin-1 and cathelicidins-2 was obviously boosted post supplementation of glutamine especially at higher levels with omega-3 suggested their efficient role in motivating the mucosal defense. These outcomes were in the same line with the higher expression levels of TJPs related genes that supported the protective role of glutamine and omega-3 against coccidia infection when administrated early in chicks’ life. Activities of Liver enzymes and kidney excretory products as uric acid and creatinine can indirectly reflect the general health status of birds. Higher serum concentrations of AST and ALT levels are considered as effective biomarkers for liver damage (Ghanima et al. 2023). Supplementation of glutamine and higher levels of omega-3 improved liver and kidney functions’ testes and hematological count before infection and restored their levels after mixed *Eimeria spp* infection. These outcomes point to a protective impact of PUFA on the integrity of hepatic and kidney cells, which may which may be related to increasing phospholipids, that are a critical constituent of integrity of cell membrane (Poorghasemi et al. 2015).

Inflammation acts as a physiological innate immune reaction to a challenge, especially at the first week of chick’s life when they take the first experiences toward their around environment, nutrition, and development changes. Conversely, severe or uncontrolled inflammations can handle the up taken nutrients to the response to the acute inflammations that cause decreased feed consumption, muscle protein deposition and muscle building, impair GIT development and week intestinal barriers and tight junctions with increase the incidence of leaky gut syndrome, increase the metabolic rate, finally resulting in high incidence of diseases and poor production (Calder 2006; Kong and Jha 2021). In our results the supplementation of glutamine alone or with different levels of omega-3 fatty acids decreased the inflammatory cytokines (IL-6 and TNF-α) and increased the anti-inflammatory cytokine IL-10 after *Eimeria spp*. challenge at 10 and 20 dpi compared with challenged non supplemented group. Previous studies illustrated that birds challenged with *Eimeria* have been shown to have higher levels of intestinal inflammatory and lower anti-inflammatory cytokines in their cecal tonsils at 6 dpi, when compared to non-challenged birds (Morris et al. 2015). In convenience with these results (Oxford and Selvaraj 2019) reported that glutamine supplementation directly after broilers’ hatch or injection in the incubated eggs had resulted in decreased expression of inflammatory cytokines *IL-6* and INF-γ and increased IL-10 after Eimeria challenge 6 dpi which indicated anticoccidial effect of early supplementation with glutamine. The main role of glutamine in early starter period in broiler is an energy source for the intestinal epithelial cells and GIT immune cells, and it plays a key role in degradation of protein, cell defense, cell restoration, and powerful antioxidant as it is a precursor of glutathione (Yu et al. 1999). Due to the immune stimulant role of glutamine in intestine, stimulation of the T cells in the mucosa will occur which is responsible for the production of cytokines as INF-γ and IL-10 that inhibit the Eimeria development (Yun et al. 2000). Furthermore, due to the sensitivity of broiler chickens to nutrient concentration and quality during the post hatching period; omega-3 fatty acids is one of the most important dietary supplementation in this period because of their potential role in reducing the intestinal inflammation, oxidative stress and improving gut health (El-Katcha et al. 2014; Jha et al. 2019). In line with our findings (Yang et al. 2006) reported an elevation in TNF-α and IL-6 after coccidial infection in broiler chickens while omega-3 supplementation alleviated this effect. Moreover, feeding broiler chickens on 10% whole flaxseed which enriched with omega-3 fatty acids triggered an increase in serum IL-6 and reduction of IL-1 and TNF-α (Aziza and Awadin 2018).

Additionally, secretory IgA in the intestinal mucosa is secreted from cecal tonsils B lymphocytes then transported from the intestinal epithelial cells into gut fluid (Corthésy 2002; Bellussi et al. 2013). SIgA antibodies are considered the main influencers in mucosal immunity as they play the key role in intestinal barrier defensing against invading pathogen through binding with invading pathogen at intestinal mucosal surfaces and neutralizing their endotoxin within epithelial cells without causing tissue damage (Brandtzaeg 2007). Herein, SIgA levels in the intestine was elevated after challenge with *Eimeria spp*. in NC in the same time supplementation with glutamine alone or glutamine combined with omega-3 further stimulated its secretion in intestinal mucosa. In consistence, Wu et al. (2022) found that glutamine supplementation increased level of IgA and IgM in broiler chickens after salmonella challenge which could be attributed to the role of glutamine as immunonutrient to mucosal B lymphocytes that secretes immunoglobulins (Fan et al. 2015). Similar results obtained by Yang et al. (Yang et al. 2006) who reported an increased in SIgA levels in the lumen of the cecum after supplementation with fish oil containing 18%EPA and 12% DHA poly unsaturated omega-3 fatty acid in broiler chickens after coccidia challenge.

Furthermore, lysozymes are lytic proteins produced the immune cell system in response to various stresses (Saurabh and Sahoo 2008). Herein, the lysozymes in the broiler serum had increased after challenge in infected control while glutamine and omega-3 supplementation decreased its level at 10 both and 20 dpi but still higher than control group. Coccidial infection in broilers proofed to generate an exaggerated different cellular and humoral immune response (Adhikari et al. 2020). Liu et al. (2017) also reported that supplementation with glutamine inhibited the lysozyme mRNA expression in the jejunum and ileum. Moreover, Wu et al. (2021) found similar decrease in serum lysozymes activity after feed supplementation with glutamine which indicated stimulation of nonspecific immunity. The motivation of lysozyme activities by glutamine indicates its role in activation of macrophages.

In the same line, Talaat (2021) proofed the important role of poly unsaturated fatty acids especially in post hatch ration to stimulate both cellular and humoral immune response against diseases. In the current study, supplementing glutamine without or with omega-3 had decreased the intestinal MPO, NO and NOS after infection with *Eimeria spp*. than infected control at both 10 and 20 day after challenge. in accordance, coccidial infection in many studies proofed to generate oxidative stress in the intestine of infected birds which manifested by increase in levels of NO and NOS (Choi et al. 2023). Glutamine supplementation in broiler rations proofed to had anti-apoptotic effects which provoked *via* death receptor-intermediated apoptotic pathway (Matés et al. 2006), throughout decreasing the production of NO by inducible NO synthase (Wu et al. 2011). Furthermore, omega-3 fatty acids supplementation exerts antioxidant effect that reduces the oxidation of tissue lipid and decrease NO and NOS. Furthermore, disease-related enzymatic oxidation markers as iNOS, and myeloperoxidase (MPO) were suppressed after supplementation with omega-3 poly unsaturated fatty acid (Sundaram et al. 2022).

Regarding the effect of supplementing broiler starter ration with glutamine and omega-3 fatty acids, in the current study, glutamine and omega-3 had decreased the severity of intestinal lesion and *Eimeria spp*. fecal oocyst shedding at 7-, 14- and 21-days post challenge and total mortality percent than infected control. From our point of view, the protective role of post hatch glutamine supplementation in improving the intestinal barriers and increasing the tight junction functional proteins, stimulating immune system through increasing the lysozymes and SIgA secretion simultaneous with activation of mucosal immune cells and cytokines may be the main cause of decreasing the severity of coccidia infection and subsequent mortality percent. Moreover, the role of supplemented omega-3 from the previous obtained results as immune stimulant; improved the anti-inflammatory cytokines, reduced the inflammatory cytokines and stimulated humoral immunity through increase SIgA secretion and lysozymes activity; antioxidant; improved the antioxidant enzymes and decreased the oxidation marker; and as a stimulant for binding barriers and intestinal tight junction function may be other cause of reducing the development of *Eimeria spp* Challenge and increased birds survival precents. Similar results obtained by Luquetti et al. (2016) who stated the effects of glutamine on performance and intestinal mucosa morphometry of broiler chickens vaccinated against coccidiosis who proofed broiler supplementation with glutamine 10 g/kg had improved birds’ response to coccidia vaccination by *Eimeria. tenella, E acervulina, E. mivati*, and *E. maxima* by improving the intestinal villus health. The protective role of natural feed stuffs containing highly amount of polyunsaturated omega-3 fatty acids against coccidia infections were first stated by Allen et al. Allen et al. (1994) who reported that inclusion of flaxseed oil or fish oil reduced lesion score in broiler chickens infected with *Eimeria Tenella*. Consistently, Yang et al. (2006) reported that broilers diets supplementation with fish oil inhibited the damaging effect caused by *E. tenella* through decreasing the blood loss and intestinal lesion score and decreasing the mortality percent.

## 5. Conclusion

The choice of good quality diets for newly hatched chicks, that fulfill and support specific nutritional needs, greatly impact their future performance especially during *Eimeria* infection. To the authors’ knowledge, it is the first time for demonstrating that dietary interventions of Glut and omega-3 combination can support broiler chickens’ performance and alleviate the damaging effects of *Eimeria* challenge. These functional ingredients exert these favorable outcomes likely by improving intestinal integrity and antioxidants potential and muscle building during the starter period. Furthermore, feeding on higher levels of Glut and omega-3 post hatching restored broilers performance during Eimeria challenge *via* subsiding excessive immune response and sustaining intestinal barrier functions over the entire rearing period. Finally, the current study recommended future reinforcement of newly hatched diets with Glut and omega-3 at the level of 1% to optimize growth and gut health prior to infection, thus reducing coccidia invasion and disease severity and maximized stress associated production of modern broiler chickens. We recommended that future studies are needed to investigate the synergistic impact of omega-3 with glutamine under other stressful conditions facing poultry farms.

## Data Availability

The data presented in this study are available upon request from the corresponding author.
